# Vitamin K2 Inhibits Hepatocellular Carcinoma Cell Proliferation by Binding to 17β-Hydroxysteroid Dehydrogenase 4

**DOI:** 10.3389/fonc.2021.757603

**Published:** 2021-11-09

**Authors:** Xin Lu, Panpan Ma, Lingyu Kong, Xi Wang, Yaqi Wang, Lingling Jiang

**Affiliations:** ^1^ Center for Prenatal Diagnosis and Genetic Diseases, Tangshan Maternal and Children Hospital, Tangshan, China; ^2^ Department of Blood Transfusion, Hebei General Hospital, Shijiazhuang, China; ^3^ Department of Oncology, Tianjin Binhai New Area Hospital of Traditional Chinese Medicine, Tianjin, China; ^4^ Department of Endocrinology, The Third Affiliated Hospital of Hebei Medical University, Shijiazhuang, China; ^5^ Department of Clinical Laboratory, Hebei Provincial Hospital of Traditional Chinese Medicine, Shijiazhuang, China; ^6^ Department of Biochemistry and Molecular Biology, The Key Laboratory of Neurobiology and Vascular Biology, Hebei Medical University, Shijiazhuang, China

**Keywords:** Vk2, HSD17B4, HCC, HepG2, STAT3

## Abstract

Our previous studies have proved that 17β-hydroxysteroid dehydrogenase 4 (HSD17B4) is a novel proliferation-promoting protein. The overexpression of HSD17B4 promotes hepatocellular carcinoma (HCC) cell proliferation. Vitamin K2 (VK2), a fat-soluble vitamin, has the function of promoting coagulation and can inhibit the progression of liver cancer. A previous study demonstrated that VK2 could bind to HSD17B4 in HepG2 cells. However, the mechanism of VK2 in inhibiting HCC cell proliferation is not clear. In this study, we investigate whether VK2 can inhibit the proliferation of HCC cell induced by HSD17B4 and the possible mechanism. We detected the effect of VK2 on HSD17B4-induced HCC cell proliferation, and the activation of STAT3, AKT, and MEK/ERK signaling pathways. We measured the effect of HSD17B4 on the growth of transplanted tumor and the inhibitory effect of VK2. Our results indicated that VK2 directly binds to HSD17B4, but does not affect the expression of HSD17B4, to inhibit the proliferation of HCC cells by inhibiting the activation of Akt and MEK/ERK signaling pathways, leading to decreased STAT3 activation. VK2 also inhibited the growth of HSD17B4-induced transplanted tumors. These findings provide a theoretical and experimental basis for possible future prevention and treatment of HCC using VK2.

## Introduction

VK2 is a fat-soluble vitamin; in addition to promoting blood coagulation, it has antitumor effects. VK2 has been shown to induce cancer cell apoptosis and suppress cancer growth and differentiation in various types of cancer cells. Recently, some studies have shown that VK2 has anticancer effects on hepatocellular cancer, breast cancer, and leukemia (1–4). Natural VK2 can inhibit HCC cell growth and induce apoptosis (5–8). In addition, VK2 has been demonstrated to exert an antiproliferative action toward a variety of cancer cells (9–12). However, the mechanisms of the inhibitory action of VK2 have not been defined.

17β-Hydroxysteroid dehydrogenase 4 (HSD17B4) is a ubiquitous oxidoreductase in mammals, with the highest levels in the liver (13, 14). Our previous studies proved that HSD17B4 is a novel proliferation-promoting gene, and overexpression or knockout of HSD17B4 can promote or inhibit the proliferation of HepG2 cells. At the same time, overexpression of HSD17B4 promotes the activity of PI3K/Akt and MEK/ERK signaling pathways, which stimulate activation of transducer and activator of transcription 3 (STAT3) and promote cell proliferation by enhancing proliferative genes expression (15, 16). The research of Otsuka et al. demonstrated that VK2 can bind to HSD17B4 in HepG2 cells (17). Whether the mechanism that VK2 inhibits the proliferation of HCC cells induced by HSD17B4 and inhibits the growth of transplanted tumors promoted by HSD17B4 by binding to HSD17B4 is not clear.

In this study, the inhibitory effect of VK2 on HCC was proved by cell and transplanted tumor experiments. We found that VK2 directly binds to HSD17B4, but does not affect the expression of HSD17B4, to inhibit the proliferation of HCC cells by inhibiting the activation of Akt and MEK/ERK signaling pathways, leading to a decrease in STAT3 activation. These findings provide a theoretical and experimental basis for possible future prevention and treatment of HCC with VK2.

## Materials and Methods

### Cell Culture and Treatment

The human hepatocellular carcinoma cell line HepG2 (MD, USA) was cultured with RPMI-1640 medium (Gibco Invitrogen Corp.) containing 10% fetal bovine serum (FBS), 100 U/ml penicillin and streptomycin. The cells were cultured in a cell incubator at 37°C, 5% CO_2_, and saturated humidity. The cell seeding density was 3 × 10^4^ cells/cm^2^. After 24 h of culture, the cells were cultured in RPMI-1640 medium without FBS for another 24 h. The HSD17B4 expression plasmid was transfected 24 h before VK2 administration.

### Plasmid Constructs and Transfection

HSD17B4 cDNA was cloned into the pLL3.7 vector (Addgene, Inc., Cambridge, MA, USA) (15). HepG2 cells (5 × 10^6^) were seeded in a six-well plate and cultured for 24 h at 37°C and 5% CO_2_. Cells were transfected when the cells have grown to about 70% confluence. The cells were washed twice with serum-free RPMI-1640, and 1 ml serum-free Opti-MEM I (Gibco; Thermo Fisher Scientific, Inc.) was added to each well. The DNA–Lipofectamine 2000 complex was prepared according to the manufacturer’s instructions (Invitrogen; Thermo Fisher Scientific, Inc.). A total of 6 μg transfection plasmid and 30 μl Lipofectamine reagent were added to each reaction. The empty vector control is indicated by “−, ” and the HSD17B4 plasmid is highlighted with a “+” below. Cells were placed in an incubator at 37°C for 6 h. The cells were cultured in Dulbecco’s modified Eagle’s medium (DMEM) containing 1% FBS for 24 h, and the transfection efficiency was determined by Western blotting.

### Small Interfering RNA Transfection

The specific small interfering RNA (siRNA) against HSD17B4 (siHSD17B4), containing 5′-GUACCUUUGUAUUUGAGGAdTdT-3′ and 5′-UCCUCAAAUACAAAGGUACdTdT-3′, and the non-specific siRNA (siNC), containing 5′-UUCUCCGAACGUGUCACGUTT-3′ and 5′-ACGUGACACGUUCGGAGAATT-3′, all were purchased from Sigma (15). Transfection was performed using the Lipofectamine reagent following the manufacturer’s instructions. Transfection was conducted after 24 h, and the cells were treated with 50 μM of VK2 for the indicated times, harvested and lysed for tests.

### Cell Proliferation Assay

Cell proliferation assays were performed using a 3-(4,5-diethylthiazol-2-yl)-5-(3-carboxymethoxyphenyl)-2-(4-sulfophenyl)-2H-etrazolium, inner salt (MTS) cell proliferation assay kit (Promega) according to the manufacturers’ recommendations. The cells were treated with MTS for 3 h before the termination of HSD17B4 plasmid incubation. The OD readings were performed to determine the number of viable cells at 490 nm. All groups were evaluated in an average of three separate wells per experiment.

### RT and Real-Time Quantitative PCR

According to the instructions, total RNA was isolated from cells using TRIzol reagent (Invitrogen). SuperScript Reverse Transcription Kit (Invitrogen) was used to synthesize cDNA, which was used as a template for real-time quantitative PCR using the SYBR Green PCR Master Mix Kit (TaKaRa). Quantitative real-time PCR was performed using the Rotor-Gene 3000 detection system (Gene Biosystems). The PCR cycle consisted of an initial denaturation step at 95°C for 30 s, followed by 40 cycles of denaturation at 95°C for 5 s, annealing at 55°C for 30 s, and extension at 72°C for 20 s. As an internal control, 18S rRNA was used for RNA template standardization. All PCRs were performed in triplicate. The relative expression level of mRNA was 2^−ΔCt^ (Ct sample −Ct control). The primer pairs are shown in **Table 1**.

**Table 1:** The primers.

Primers for PCR	Sequence
**For gene expression:**	
18SrRNA	5′-ATCCCTGAAAAGTTCCAGCA-3′
(NM_022551)	5′-CCCTCTTGGTGAGGTCAATG-3′
HSD17B4	5′-TTGGGCCGAGCCTATGC-3′
(NM_ 000414)	5′-CCCCTCCCAAATCATTCACA-3′

### Western Blotting

Cells are lysed in lysis buffer A for cytoplasmic protein extraction, and then, the cells were used for nucleoprotein extraction in lysis buffer B (15). After the lysates were incubated on ice for 30 min, nuclear protein was obtained by centrifugation at 8,000 *g* for 10 min, or cytoplasmic protein was obtained by centrifugation at 12,000 g for 20 min. The supernatant was used for testing. The same amount of proteins (100–200 μg) were separated by 10% sodium dodecyl sulfate–polyacrylamide gel electrophoresis (SDS-PAGE) and electro-transferred to polyvinylidene fluoride (PVDF) membrane. The membrane was sealed with 5% skim milk powder for 1 h at room temperature and incubated overnight at 4°C with primary antibodies: rabbit anti-HSD17B4 (Homemade), 1:2,000 (Jiang et al., 1996); rabbit anti-cyclin D1 (Cell Signaling, Inc., catalog no. 2922S), 1:1,000; rabbit anti-PCNA (Cell Signaling, Inc., catalog no. 13110), 1:1,000; mouse anti-β-actin (Santa Cruz Biotechnology, Inc., catalog no. sc-47778), 1:1,000; rabbit anti-p-STAT3 (Santa Cruz Biotechnology, Inc., catalog no. sc-135649), 1:500; rabbit anti-STAT3 (Santa Cruz Biotechnology, Inc., catalog no. sc-482), 1:500; rabbit anti-p-Akt (Santa Cruz Biotechnology, Inc., catalog no. sc-7985), 1:500; rabbit anti-Akt (Santa Cruz Biotechnology, Inc., catalog no. sc-8312), 1:500; rabbit anti-p-MEK (Santa Cruz Biotechnology, Inc., catalog no. sc-101733), 1:500; rabbit anti-MEK (Santa Cruz Biotechnology, Inc., catalog no. sc-9259), 1:500; rabbit anti-p-ERK (Santa Cruz Biotechnology, Inc., catalog no. sc-23759), 1:500; and rabbit anti-ERK (Santa Cruz Biotechnology, Inc., catalog no. sc-292838), 1:500. After incubation with appropriate fluorescent secondary antibodies at room temperature for 1 h, the anti-antigen complexes were imaged using chemiluminescence Plus Protein Western blot assay kit (Santa Cruz Biotechnology). The expressions of various genes were expressed in relative grayscale, and β-actin was used as the protein loading control.

### Enzyme-Linked Immunosorbent Assay

HepG2 cells with HSD17B4 overexpression were treated with different concentrations of VK2 for 48 h. The cell lysates interacted with anti-HSD17B4 antibody; the precipitate was collected for the determination of the content of VK2 that bound to HSD17B4 in cells. Subsequently, a cell lysates assay for anti-VK2 antibody was performed by commercial ELISA kit (BlueGene Biotech) according to the manufacturer’s instructions. The optical density (OD) was measured at 450 nm. The results were expressed as pg/ml according to a calibrator curve.

### Nude Models and Treatments

In order to test the inhibitory effects of VK2 on tumor growth *in vivo*, 2 × 10^6^ transduced HepG2 cells were injected into BALB/c nude mice aged 4 weeks. Six weeks post-inoculation, the animals were sacrificed, and the volume of the tumor was recorded.

### Statistical Analysis

The results were expressed as mean ± standard deviation (SD). Statistical differences among groups were analyzed using one-way ANOVA. At the p < 0.05 level, the difference was considered statistically significant. All statistical analyses were performed using SPSS 13.0 software.

## Results

### VK2 Suppressed HepG2 Cells Proliferation Induced by HSD17B4

**Figure 1:**
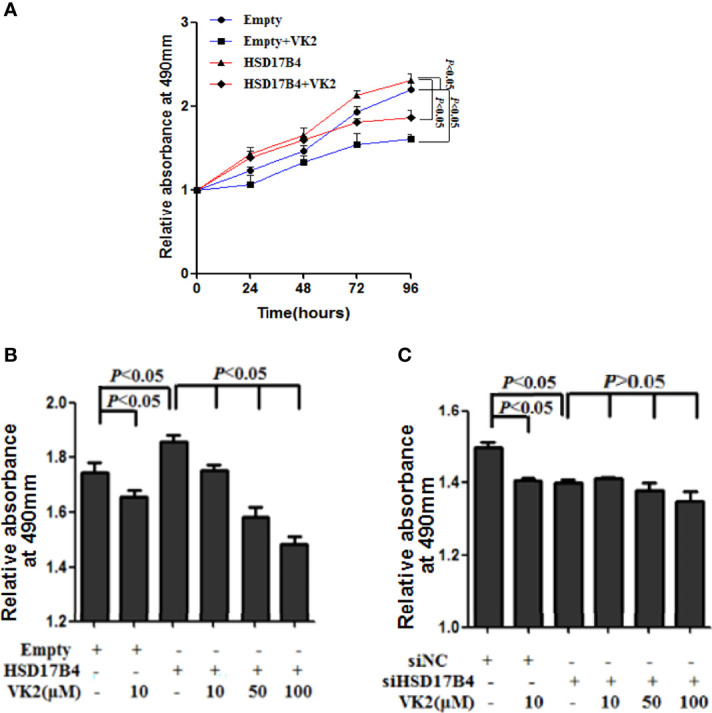
VK2 suppressed HepG2 cells proliferation induced by HSD17B4. **(A)** HepG2 cells were treated with or without vitamin K2 (50 μM) for 0, 24, 48, 72, and 96 h after transfection with HSD17B4 overexpression (HSD17B4) or control (empty) plasmids. **(B)** HepG2 cells were treated with different concentrations of vitamin K2 (0, 10, 50, and 100 μM) for 48 h after transfection with HSD17B4-overexpression plasmids (HSD17B4) or control plasmids (empty), and **(C)** with specific (siHSD17B4) or non-specific (siNC) siRNA sequences against HSD17B4. The proliferation of the treated cells was assessed by MTS and indicated as relative absorbance at 490 nm. The results are the mean ± SD from three independent experiments performed in triplicate.

To test the effect of VK2 on HCC cells proliferation induced by HSD17B4, we measured the activation of cells by MTS assay. VK2 (50 μM) showed a time-dependent inhibition on the proliferation of HepG2 cells with HSD17B4 overexpression (**Figure 1A**). It was statistically significant from 24 h (p < 0.05).

To further verify that VK2 can directly inhibit proliferation of HCC cells with HSD17B4 overexpression, we measured the activation of cells that were transfected with expression plasmid of HSD17B4 or siHSD17B4 to increase or knock down HSD17B4 expression by MTS assay. VK2 showed a dose-dependent inhibition on the proliferation in HSD17B4 overexpressed HepG2 cells (**Figure 1B**). However, there was no significant difference in a dose-dependent inhibition on transfection with siHSD17B4 (**Figure 1C**). These results showed that VK2 could suppress cell proliferation induced by HSD17B4, and the inhibition of proliferation by VK2 requires the presence of HSD17B4.

### VK2 Bound to HSD17B4 Directly and Did not Affect its Expression

**Figure 2:**
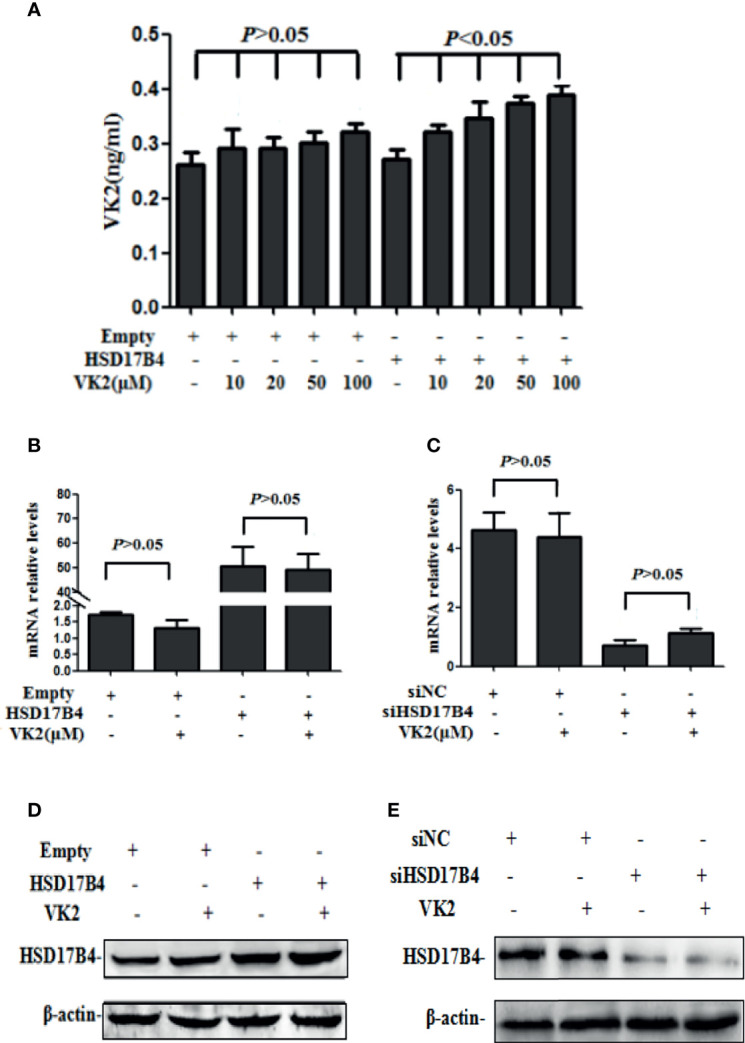
VK2 bound to HSD17B4 directly and did not affect its expression. HepG2 cells were treated with different concentrations of vitamin K2 (0, 10, 20, 50, and 100 μM) for 48 h after transfection with HSD17B4-overexpression (HSD17B4) or control (empty) plasmids. **(A)** Vitamin K2 levels in anti-HSD17B4 immunoprecipitates of lysates from above cells were assayed by ELISA to indicate the combination of VK2 and HSD17B4 protein. HepG2 cells were treated with vitamin K2 (50 μM) for 48 h after transfection with HSD17B4-overexpression (HSD17B4) or control (empty) plasmids and with specific (siHSD17B4) or non-specific (siNC) siRNA sequences against HSD17B4. The **(B**, **C)** mRNA and **(D**, **E)** protein expression of above groups were assayed by qRT-PCR and Western blotting. The results are the mean ± SD from three independent experiments performed in triplicate.

It has been reported that VK2 can bound to HSD17B4 in HepG2 cells (17). To further prove that VK2 can bind to HSD17B4 directly, we used anti-HSD17B4 antibody to precipitate HSD17B4 in the lysates of HepG2 cells and then detect VK2 content in the precipitate. The VK2 concentrations in HepG2 cells were assayed by ELISA. HepG2 cells were transfected with the empty or HSD17B4 expression plasmid. Then, these cells were treated with VK2. As shown in **Figure 2A**, VK2 showed a dose-dependent increase in the cells transfected with HSD17B4 expression plasmids, but there was no significant increase in VK2 on the HepG2 cells transfected with the empty plasmid. These results demonstrated that VK2 binds to HSD17B4 to exert its inhibitory effect on HepG2 cells.

Furthermore, we tested the change in HSD17B4 expression in mRNA and protein by VK2 treatment. VK2 did not affect the HSD17B4 expression of mRNA and protein in HepG2 cells by transfecting with a HSD17B4 expression plasmid (**Figures 2B, D** **)**. At the same time, VK2 also did not affect the mRNA and protein expression of HSD17B4 by siRNA to knockdown HSD17B4 expression (**Figures 2C, E** **)**. These results indicated that VK2 bound to HSD17B4 directly and did not affect the expression of HSD17B4.

### VK2 Downregulated the HSD17B4-Induced Expression of Phosphorylated STAT3 Gene and Proliferation in HepG2 Cells

**Figure 3:**
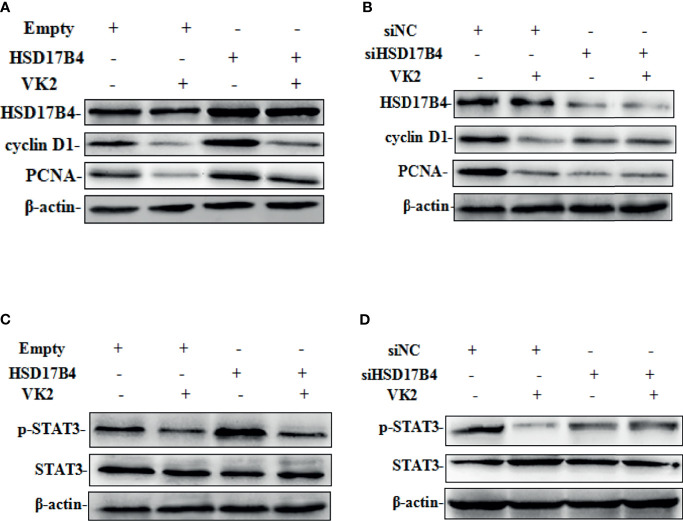
VK2 downregulated the HSD17B4-induced expression of phosphorylated STAT3 gene and proliferation in HepG2 cells. HepG2 cells were treated with vitamin K2 (50 μM) for 48 h after transfection **(A**, **C)** with HSD17B4-overexpression (HSD17B4) or control (empty) plasmids, and **(B**, **D)** with specific (siHSD17B4) or non-specific (siNC) siRNA sequences against HSD17B4. The proteins of cyclinD1, PCNA, STAT3, and p-STAT3 were assayed by Western blot.

Our recent studies have found that upregulation of HSD17B4 promoted HepG2 cell proliferation by enhancing cyclin D1 and proliferating cell nuclear antigen (PCAN) expression (16). To determine whether VK2 could reduce the HSD17B4-induced overexpression of proliferation genes, we investigated the expression of cyclin D1 and PCNA on the cells that were transfected with HSD17B4 expression plasmids or siHSD17B4. As shown in **Figure 3**, HSD17B4 overexpression increased the protein levels of cyclin D1 and PCNA, and VK2 not only suppressed the expression of cyclin D1 and PCNA on cells that were transfected with empty plasmids but also suppressed their increased expression induced by HSD17B4 overexpression (**Figure 3A**). However, there was no significant decrease in the expression of cyclin D1 and PCNA on the HepG2 cells that were transfected with siHSD17B4 to knockdown HSD17B4 expression (**Figure 3B**). These results indicated that VK2 inhibited the proliferation genes expression of HSD17B4-increased protein of cyclin D1 and PCNA in HepG2 cells.

To further investigate the mechanism by which VK2 suppressed proliferation induced by HSD17B4 overexpression in HepG2 cells, we detected the activation of STAT3 pathway that was directly related to proliferation of HCC cells. HSD17B4 overexpression increased the phosphorylation level of STAT3, and VK2 not only suppressed phosphorylation of STAT3 on cells that were transfected with the empty plasmids but also suppressed their increased activation by induced HSD17B4 overexpression (**Figure 3C**). However, there was no significant decrease in the phosphorylation of STAT3 on the HepG2 cells that were transfected with siHSD17B4 to knockdown HSD17B4 expression (**Figure 3D**). These results indicated that VK2 suppressed expression of proliferation genes by downregulation of HSD17B4-increased phosphorylation of STAT3 in HepG2 cells.

### VK2 Downregulated HSD17B4-Induced Phosphorylation of Akt and MEK/ERK in HepG2 Cells

**Figure 4:**
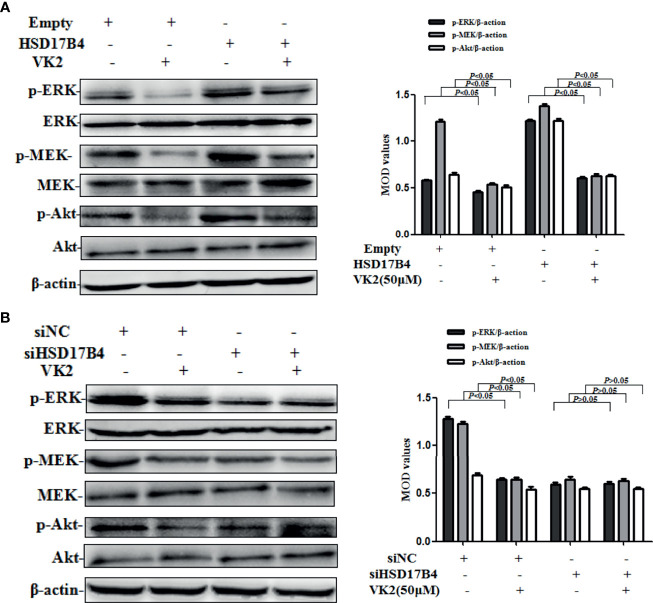
VK2 downregulated HSD17B4-induced phosphorylation of Akt and MEK/ERK in HepG2 cells. HepG2 cells were treated with vitamin K2 (50 μM) for 48 h after transfection **(A)** with HSD17B4-overexpression (HSD17B4) or control (empty) plasmids, and **(B)** with specific (siHSD17B4) or non-specific (siNC) siRNA sequences against HSD17B4. The proteins of Akt and p-Akt, MEK and p-MEK, and ERK and p-ERK were assayed by Western blotting and the corresponding quantitative data. Three independent experiments were performed in triplicate.

STAT3 phosphorylation can be mediated through the activation of upstream signaling pathway, including Akt and MEK/ERK, that were involved in the proliferation of HCC cells. To further investigate whether VK2 was involved in the downregulation of HSD17B4-increased activation of upstream signaling pathway of STAT3 in HepG2 cells, we detected the phosphorylation of Akt and MEK/ERK. HSD17B4 overexpression increased the phosphorylation levels of Akt, MEK, and ERK, and VK2 not only suppressed their phosphorylation on cells that were transfected with the empty plasmids but also suppressed their increased activation by induced HSD17B4 overexpression (**Figure 4A**). However, there was no significant decrease in the phosphorylation of Akt, MEK, and ERK on the HepG2 cells that were transfected with siHSD17B4 to knockdown HSD17B4 expression (**Figure 4B**). These results indicated that VK2 inhibited the downregulation of HSD17B4-induced phosphorylation of Akt and MEK/ERK in HepG2 cells.

### VK2 Inhibited the Growth of Xenograft Tumor in Nude Mice Injected With HSD17B4-Overexpression HepG2 Cells

**Figure 5:**
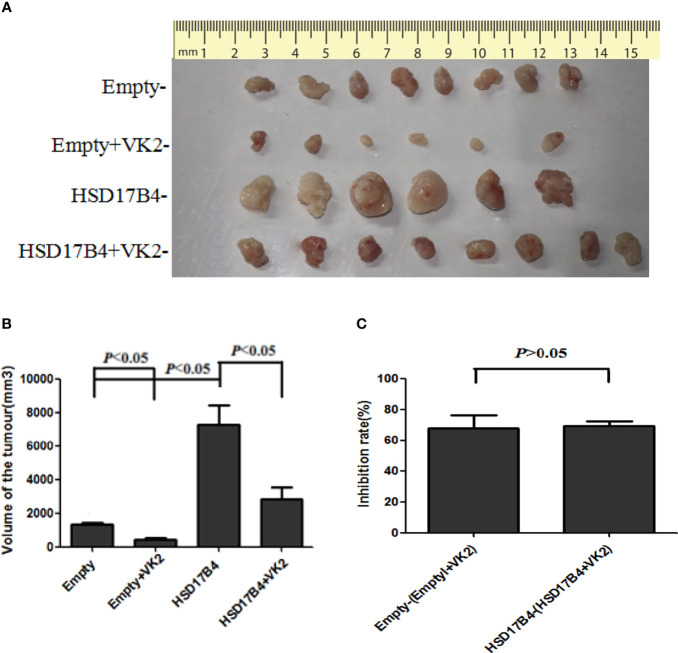
VK2 inhibited the growth of xenograft tumor in nude mice injected with HSD17B4-overexpression HepG2 cells. HepG2 cells were treated with or without vitamin K2 (50 μM) for 48 h after transfection with HSD17B4-overexpression (HSD17B4) or control (empty) plasmids. The treated HepG2 cells were subcutaneously injected into the axillary of nude mice to grow for 6 weeks. The xenografts were harvested from groups of empty (n = 8), HSD17B4 (n = 6), empty + VK2 (n = 6), and HSD17B4 + VK2 (n = 6). Tumor sizes were shown in **(A)**. The mean volume of tumors is plotted in **(B)**. The inhibition rate of tumor growth by VK2 is shown in **(C)**. The data are presented as mean ± SD.

Typically, a single nodular tumor was found mostly localized within the injected site and was observed in animals implanted with HepG2 cells. The xenograft tumors are shown in **Figure 5A**. To compare the differences in tumor volume, we measured the volume of the tumor (**Figure 5A**) and compared the mean volume of tumors in each group (**Figure 5B**). Evidently, the tumors volumes were increased in HSD17B4 plasmids transfection but were reduced significantly by VK2 treatment. To further preclude the interaction of VK2 with HSD17B4, the inhibitory rate of tumor growth by VK2 showed that there was no significant difference between control (empty mean/empty + VK2 mean) and HSD17B4 overexpression (HSD17B4 mean/HSD17B4 + VK2 mean) (**Figure 5C**). Thus, the results exhibited that VK2 can inhibit the tumor growth by HSD17B4 overexpression.

## Discussion

VK2 is a common fat-soluble vitamin that is involved in a variety of metabolism, such as blood coagulation and bone metabolism (1, 2). In recent years, its antitumor effect has received extensive attention (3, 4). Many studies both *in vitro* and *in vivo* have demonstrated that VK2 inhibits cancer growth and induces apoptosis and differentiation of various types of cancer cells, including leukemia (18), melanoma (19), and HCC cells (5, 6). Recent studies have shown that vitamin K plays a role in the prognosis of HCC (20, 21). VK2 is a safe and effective therapy for inhibiting HCC recurrence and improving prognosis (22). In this study, our results were consistent with most reports. VK2 not only inhibited the tumor growth on nude mice bearing HepG2 cells (**Figure 5**) but also suppressed the proliferation of HepG2 cells by inducing HSD17B4 expression.

HSD17B4 is an important oxidoreductase in mammals (13, 14). Recently, HSD17B4 has been shown to be expressed highly in prostate cancer cells (23) and prostate tissues from prostate cancer patients (24–26), and the level of expression was related to the progression of cancer (24). Our previous studies demonstrated that the expression of HSD17B4 was increased in the tissue of HCC (16), and HSD17B4 can promote the proliferation of HCC cells (15). Otsuka et al. found that newly synthesized biotinylated VK2 could affect the activity of HSD17B4 (17). Under our experimental conditions, we also proved that VK2 binds to HSD17B4 to exert its inhibitory effect. At the same time, our results indicate that VK2 does not affect the expression of HSD17B4.

VK2 is an essential clinical drug for patients with liver cancer. In our experiment, it was proved that the expression of HSD17B4 was elevated in HCC patients, and the high expression of HSD17B4 promoted tumor proliferation (15, 16). VK2 could inhibit HSD17B4-induced HCC cell proliferation. Therefore, the supplementation of VK2 in HCC patients is of great significance, and this study provides a new theoretical basis for the application of VK2 in the treatment of HCC.

Many studies have proved that STAT3 as a nuclear transcription factor promoting tumor development plays an important role in the proliferation of liver cancer (27, 28), and inhibition of STAT3 activity can inhibit the proliferation of HCC cells (29–31). Previous studies have suggested that the antitumor mechanism of VK may be related to the inhibition of signaling pathway activity. VK2 promotes apoptosis of HCC cells by inhibiting MEK signaling pathway (32), and VK3 can inhibit STAT3 activation (33). Our previous studies have confirmed that HSD17B4 overexpression resulted in enhanced activation of STAT3 (15). In this study, VK2 directly binds to HSD17B4 to inhibit the proliferation of HCC cells by inhibiting the activation of Akt and MEK/ERK signaling pathways, leading to decreased STAT3 activation (**Figure 5**).

**Figure 6:**
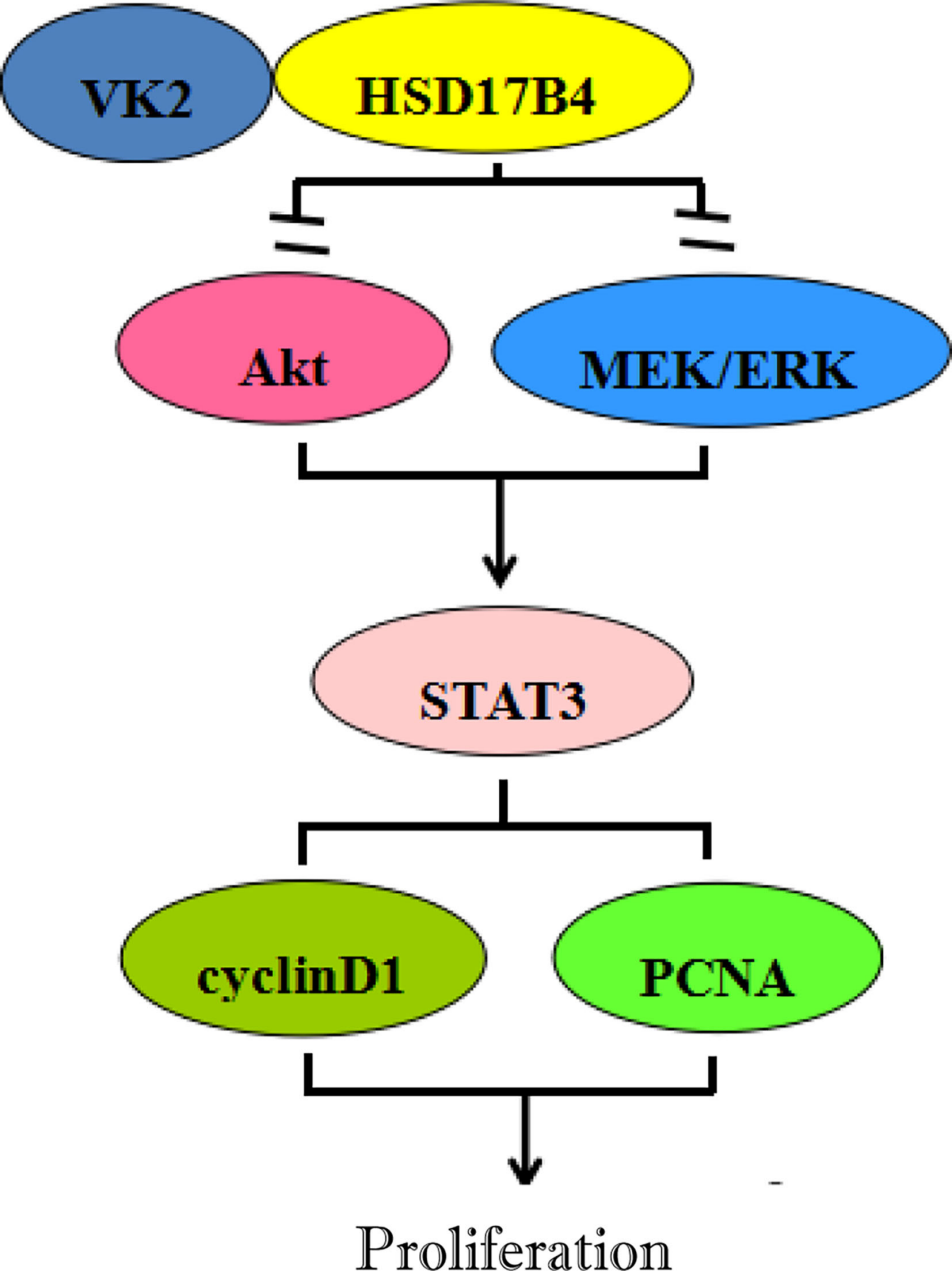
Inhibitory effects of VK2 on HSD17B4-induced proliferation of HCC cells.

In summary, VK2 directly binds to HSD17B4, but does not affect the expression of HSD17B4, to inhibit the proliferation of HCC cells by inhibiting the activation of Akt and MEK/ERK signaling pathways. VK2 can decrease STAT3 activation, then decrease the expression of proliferation gene cyclin D1 and PCNA, eventually leading to proliferation suppression of HCC cells (**Figure 6**).

## Data Availability Statement

The original contributions presented in the study are included in the article/supplementary material. Further inquiries can be directed to the corresponding author.

## Author Contributions

LJ is responsible for experiment design and article revision. XL, LK, and PM are responsible for experiment implementation, manuscript writing, and article editing. All authors contributed to the article and approved the submitted version.

## Funding

This study was funded by Science Foundation of Hebei province, China (20210400).

## Conflict of Interest

The authors declare that the research was conducted in the absence of any commercial or financial relationships that could be construed as a potential conflict of interest.

## Publisher’s Note

All claims expressed in this article are solely those of the authors and do not necessarily represent those of their affiliated organizations, or those of the publisher, the editors and the reviewers. Any product that may be evaluated in this article, or claim that may be made by its manufacturer, is not guaranteed or endorsed by the publisher.
